# Retracted: Cure of Chronic Viral Infection and Virus-Induced Type 1 Diabetes by Neutralizing Antibodies

**DOI:** 10.1155/2016/2747402

**Published:** 2016-01-21

**Authors:** Journal of Immunology Research

The paper titled “Cure of Chronic Viral Infection and Virus-Induced Type 1 Diabetes by Neutralizing Antibodies” [1] has been retracted as it was found that the paper was unintentionally published twice by the journal's former publisher. The version of record of this paper is available at http://dx.doi.org/10.1080/17402520600800721.
